# Comprehensive assessment on the applications of oncolytic viruses for cancer immunotherapy

**DOI:** 10.3389/fphar.2022.1082797

**Published:** 2022-12-08

**Authors:** Richard Kolade Omole, Oluwaseyi Oluwatola, Millicent Tambari Akere, Joseph Eniafe, Elizabeth Oladoyin Agboluaje, Oluwafemi Bamidele Daramola, Yemisi Juliet Ayantunji, Temiloluwa Ifeoluwa Omotade, Nkem Torimiro, Modupe Stella Ayilara, Oluwole Isaac Adeyemi, Olubusola Sajanat Salinsile

**Affiliations:** ^1^ Department of Microbiology, Obafemi Awolowo University, Ile-Ife, Nigeria; ^2^ Microbiology Unit, Department of Applied Sciences, Osun State College of Technology, Esa-Oke, Nigeria; ^3^ Department of Molecular Medicine, Morsani College of Medicine, University of South Florida, Tampa, FL, United States; ^4^ Department of Immunology, Moffit Cancer Center, Tampa, FL, United States; ^5^ Department of Medicinal and Biological Chemistry, University of Toledo, Toledo, OH, United States; ^6^ Department of Microbiology and Immunology, Louisiana State University Health Sciences Center, Shreveport, LA, United States; ^7^ Department of Pharmaceutical and Biomedical Sciences, University of Georgia, Athens, GA, United States; ^8^ Advanced Space Technology Applications Laboratory, Cooperative Information Network, National Space Research and Development Agency, Ile-Ife, Nigeria; ^9^ Food Security and Safety Focus Area, Faculty of Natural and Agricultural Sciences, North-West University, Mmabatho, South Africa; ^10^ Department of Pharmacology, Faculty of Pharmacy, Obafemi Awolowo University, Ile-Ife, Nigeria

**Keywords:** cancer, viro-immunotherapy, oncolytic virus, tumor microenvironment, treatment, anti-cancer, immunology, targeted killing

## Abstract

The worldwide burden of cancers is increasing at a very high rate, including the aggressive and resistant forms of cancers. Certain levels of breakthrough have been achieved with the conventional treatment methods being used to treat different forms of cancers, but with some limitations. These limitations include hazardous side effects, destruction of non-tumor healthy cells that are rapidly dividing and developing, tumor resistance to anti-cancer drugs, damage to tissues and organs, and so on. However, oncolytic viruses have emerged as a worthwhile immunotherapeutic option for the treatment of different types of cancers. In this treatment approach, oncolytic viruses are being modeled to target cancer cells with optimum cytotoxicity and spare normal cells with optimal safety, without the oncolytic viruses themselves being killed by the host immune defense system. Oncolytic viral infection of the cancer cells are also being genetically manipulated (either by removal or addition of certain genes into the oncolytic virus genome) to make the tumor more visible and available for attack by the host immune cells. Hence, different variants of these viruses are being developed to optimize their antitumor effects. In this review, we examined how grave the burden of cancer is on a global level, particularly in sub-Saharan Africa, major conventional therapeutic approaches to the treatment of cancer and their individual drawbacks. We discussed the mechanisms of action employed by these oncolytic viruses and different viruses that have found their relevance in the fight against various forms of cancers. Some pre-clinical and clinical trials that involve oncolytic viruses in cancer management were reported. This review also examined the toxicity and safety concerns surrounding the adoption of oncolytic viro-immunotherapy for the treatment of cancers and the likely future directions for researchers and general audience who wants updated information.

## 1 Introduction

Cancer is a global-leading cause of death which accounted for estimated 10 million deaths in the year 2020, meaning that cancer is responsible for the death of one in every six dead persons in the world (Ferlay et al., 2020; World Health Organization, 2022). This death toll is expected to continue to rise with a predicted 13.1 million deaths in the year 2030 alone (Bray et al., 2018; Ferlay et al., 2020; World Health Organization, 2022). To show how grievous cancer is, the deaths caused by tuberculosis, malaria and HIV/AIDS put together is still lesser than the number of deaths caused by cancer alone (Bray et al., 2018; Ferlay et al., 2020). The World Health Organization has listed lung, colon, stomach and breast cancers as the most common in terms of new cases and the most common causes of death due to cancer (World Health Organization, 2022). In the developed countries of the world like the United States of America, cancer is the second disease with the most number of deaths. The number of new cases and deaths in 2022 alone has been predicted to be 1,918,030 and 609,360, respectively, while about 350 deaths have been estimated to happen for lung cancer every day in the year 2022 (Siegel et al., 2022). Between 2017 and 2019, about 167,000 people died of cancer in the United Kingdom; that’s 89,200 males and 78,000 females. This statistics from the UK between 2017 and 2019 showed that about 460 people died daily and one person died every 4 min (Cancer Research UK, 2019).

In the developing nations, cancer is among the top three causes of deaths in adults (Wong et al., 2018). Prior this time, in 2002 precisely, about 6.7 million deaths were recorded to have been caused by cancer, but the death toll in sub-Saharan Africa accounted for less than 5% of these deaths (Torre et al., 2015). Exactly 10 years ago (2012) in the sub-Saharan Africa, new cases of cancer were estimated to be 626, 400 and number of deaths were recorded to be 447,700 (Plummer et al., 2016; Sharma et al., 2022). However, the cancer death toll in sub-Saharan Africa has continued to rise (Olaleye and Ekrikpo, 2017; Sharma et al., 2022). The continuous rise in the incidence and mortality of cancer in sub-Saharan Africa has been linked to late presentation and diagnosis, poor access to treatment facilities and poor outcomes in cases where access to treatment was granted. It has been estimated that 80%–90% of advanced stage cancer cases result to death due to insufficient access to treatment facilities and necessary infrastructure (Bray et al., 2018; Ferlay et al., 2020).

In Nigeria, there are 102,000 new cases of cancer every year, while about 72,000 people die of cancer annually (Federal Ministry of Health, 2018; Fatiregun et al., 2020). In a more recent study, the incidence of cancer in Nigeria was estimated to be between 118,101 and 131,911, with death toll ranging between 74,234 and 83,857 (Sharma et al., 2022). The pattern of cancer incidence in Nigeria has continued to increase, but the cancer data collection in Nigeria is poor (Federal Ministry of Health, 2018). Hence, there is not much information on the annual cancer mortality trends and patterns, particularly for different states in Nigeria. Cancer, among other complex diseases, has emerged to require critical health care. There is need to direct global efforts to reduce the number of new cancer cases and provide adequate treatment to reduce the mortality rates as fast as possible, particularly in sub-Saharan Africa (Sharma et al., 2022).

### 1.1 Conventional approaches to the treatment of cancer and their limitations

Over the years, different approaches have been used to treat cancer with some level of successes achieved, however, not without their limitations. It is interesting to know that more than 50% of all global clinical trials in the world are targeted on cancer therapy (Abbas and Rehman, 2018; Ferlay et al., 2020). Some of the prominent conventional methods used for treating various forms of cancer include surgical operations, radiotherapy with x-rays and chemotherapy which involves the use of anti-cancer drugs either to cure cancer, lessen the severity of the symptoms or extend the life of the patient (Arruebo et al., 2011; Mondal et al., 2014). Chemotherapy could be used singly or in synergy with radiotherapy, and it has been reported to be the most globally used and most effective treatment in cancer therapy (El-Hussein et al., 2020). Chemotherapeutic drugs target and destroy the tumor cells by the production of reactive oxygen species, a phenomenon tagged genotoxicity (El-Hussein et al., 2020; Debela et al., 2021). However, surgical operation is still the most effective treatment therapy for the removal of cancers at the early stage of disease development (El-Hussein et al., 2020; Debela et al., 2021).

Some of the limitations or drawbacks of these conventional cancer therapies include damage to non-tumor healthy cells, tissues or organs, which is very common with radiotherapy. Almost all the available chemotherapeutic anti-cancer drugs have negative impact on cells that are dividing and developing swiftly, but that are not cancerous cells (El-Hussein et al., 2020; Debela et al., 2021). However, the main issue with the chemotherapeutic approach is the inability of an anti-cancer drug which was once effective in suppressing some cancer cells to become ineffective against the same cancer cells; a phenomenon referred to as drug resistance. This development of drug resistance by cancer cells has been attributed to increase in drug efflux and decrease in drug uptake (Shapira et al., 2011; El-Hussein et al., 2020). Other drawbacks associated with chemotherapy are; fast drug metabolism, dangerous side effects, absence of specificity and difficulty in selection of dosage (Mondal et al., 2014). The result from surgery cannot be effective at the advanced stage and it is unfortunate that only few registered cases are discovered at the early stage of disease development, with over 60% discovered at the advanced stage (Damyanov et al., 2018). The success of the surgical operation is also dependent on the skillfulness of the medical surgeon, but in cases where there is high standard surgery, some micro-tumor cells are not discoverable during the surgery and *via* diagnostic tools too, so, such cells could progress in the future to become full blown tumor cells. Some other limitations associated with surgical removal of cancer include complications from poor anesthesia, infections, cancer cells distributed in the blood flow and immune system suppression. The last two limitations have been linked to metastases distribution in the body of patients who have undergone surgery (Demicheli et al., 2008; El-Hussein et al., 2020).

In recent times, several approaches are being developed to improve on the various limitations of conventional therapy which include; use of natural antioxidants and nanoparticles, targeted drug therapy (monoclonal antibodies, small molecule inhibitors and ablation cancer therapy), stem cell therapy, sonodynamic therapy, chemodynamic therapy, ferroptosis-based therapy and gene therapy (Abbas and Rehman, 2018; Debela et al., 2021). These approaches have focused on producing efficient and safe cancer therapies. Among these, gene therapy stands out in preventing cancer progression by the insertion of a defective gene into the genome to lyse the tumor cells directly. Gene therapy includes the use of oncolytic viruses, Rexin-G, Kymriah, Zalmoxis, Genicine, among many others being developed (Abbas and Rehman, 2018; Debela et al., 2021).

### 1.2 The role of oncolytic viruses in cancer immunotherapy

An integral quality of viruses is their ability to selectively replicate and induce cytopathic effects; these qualities, among others have made them well suited for cancer immunotherapy. The viral genome is easily adaptable to changes that boost their affinity (viral tropism) for neoplastic cells (Kaufman et al., 2013; Engeland, 2020). Oncolytic viruses (OVs) are gaining popularity in tumor treatment because they elicit T cell responses and in turn anti-tumor immunity; they are therefore immunogenic in nature, hence their ability to trigger an anti-tumor immune response (Fukuhara et al., 2016; Guo et al., 2017). Following the success of immunotherapy using immune checkpoint inhibitors (Gujar et al., 2018; Vijayakumar et al., 2020), oncolytic viral immunotherapy may represent the next significant advancement in the fight against cancer. Tumor cells through their manipulation thrive in the “harsh environment” of the immune system. They minimize the expression of their neo-antigens and prevent infiltration of effector cells to the tumor bed, paralyzing innate and adaptive immune responses (Russell et al., 2012; Engeland, 2020). Studies have shown that the tumor microenvironment (TME) reconditions their environment to escape immunosurveillance and promote tumor growth (Jiang et al., 2019; Wang et al., 2022).

The principle by which oncolytic viruses act are multimodal and provide a strong rationale for their use in cancer immunotherapy (Lichty et al., 2014; Engeland, 2020; Heidbuechel and Engeland, 2021). They possess activated cell signaling pathways that encourage tumor cell proliferation, while promoting the growth and propagation of viruses within the malignancy (Engeland, 2020; Zeng et al., 2021). Interestingly, tumor cells are limited in their ability to defensively respond to viral infections compared to normal tissues (Lichty et al., 2014; Engeland, 2020). OVs utilize this limitation to their advantage by targeting and destroying the tumor. Their restriction to the tumor site stems from their dependence on the hallmarks of cancer (tumor-specific changes) including defects in antiviral response and altered receptor expression, hence, healthy tissues are unharmed (Engeland, 2020; Heidbuechel and Engeland, 2021).

In this review article, we examined the relevance of oncolytic viro-immunotherapy in the treatment of cancer to improve on the setbacks of the conventional treatment therapies. The mechanism behind this promising and novel anti-cancer approach was also presented in details. With comprehensive explanation, we described the different classes of oncolytic viruses that have found their application in the treatment of different forms of cancers, both at the experimental and clinical trial phases (Tables 1–4). The possible safety and toxicity concerns surrounding the application of oncolytic viro-immunotherapy in cancer treatment were considered and the areas where research efforts should be channeled in the future to better fortify the resource of oncolytic viruses as immunotherapeutic agents in the global fight against cancer were clearly presented as well.

**TABLE 1:** Summary of herpesviruses implicated as oncolytic viro-immunotherapeutic agents against cancer.

Oncolytic virus	Nucleic acid	Any genetic modification?	Immune Cells Involved	Cancer type/location	Delivery mode	Outcome	Reference
Herpes Simplex Virus (HSV)	dsDNA	IL-4 HSV; Both copies of the γ_1_34.5 gene were supplanted with murine genes encoding IL-4 and IL-10	Macrophages	Glioma/Brain	Intratumoral	IL-4 HSV increased the survival of glioma-bearing mice. Whereas the IL-10 HSV was unable to modulate the survival of these mice	Andreansky et al. (1998)
HSV-1	dsDNA	△N146; a selective editing of the γ_1_34.5 gene, to bear only its 147 to 263 amino acids, was carried out.	T-cells	Breast carcinoma/right flanks	Intratumoral	△N146 significantly reduced the growth of primary tumors	Liu and He, (2019)
HSV-1	dsDNA	Thymidine kinase-negative mutation	Lymphocytes (which indicates that the adaptive immune system is at play)	Gliomas/brain	Intraneoplastic	Significant growth inhibition of tumor in virus-treated mice when compared to the control-treated.	Martuza et al. (1991)
HSV-1	dsDNA	γ_1_34.5 mutant expressing both sub units of mIL-12	T lymphocytes and macrophages	Neuroblastoma/brain	Intratumoral	Median survival of the mutant virus-treated mice was higher than that of those treated with another mutant virus, which lacks any cytokine gene insert	Parker et al. (2000)
HSV-1	dsDNA	VC2; possesses a deletion of 38 amino acids in the N terminus of the viral envelope glycoprotein K and an additional deletion of amino acids 4 to 22 of a second envelope.	CD8^+^ T-cells	Melanoma/dermis of the dorsal left dorsal pinna (ear)	Intratumoral	Significant survival of tumor-engrafted VC2-treated mice over the control treated ones	Uche et al. (2021)
HSV-1	dsDNA	G47△; possesses a deletion of the α47 gene from its γ34.5 deficient HSV-1 vector, G207.	T-cells	Human melanoma cell lines; Glioma	Intraneoplastic (*in vivo*)	Increased MHC class 1 expression in virus treated cells as well as rapid tumor cell-death. Reduction in tumor growth human xenograft mice model.	Todo et al. (2001)
HSV-1	dsDNA	Oncolytic HSV G47△; (ICP6^−^, γ34.5^−^, α47^−^)	Not mentioned	Glioblastoma	Intratumoral	Prolonged the survival of mice with intracerebral tumors generated by glioblastoma-derived cancer stem-like cells (GBM-SC)	Wakimoto et al. (2009)
HSV-1	dsDNA	rRp450/CPA; ICP6^−^ and expresses a prodrug enzyme for cyclophosphamide (CPA)	No significant inflammatory response	Solid tumors (sarcomas)	Intravenous	rRp450/CPA is safe for use as a potential anticancer therapeutic	Currier et al. (2008)
					Intracranial		
					Intraperitoneal		
HSV-1	dsDNA	Mesenchymal stem cells-loaded HSV variants (MSC-oHSV)	T lymphocytes	Melanoma/Brain	Intracarotid	The use of MSC as oHSV carriers helped in the tracking and killing of metastatic melanoma cells in the brain.	Du et al. (2017)
HSV-1	dsDNA	MSC-oHSV, sECM-encapsulated MSC-oHSV, and MSC-oHSV-TRAIL (tumor necrosis factor-related apoptosis-inducing ligand) variants	Not mentioned	Glioblastoma Multiforme (GBM)	Intratumoral	MSC-oHSV-TRAIL variant significantly increased the median survival time of the mice as compare to other variants.	Duebgen et al. (2014)
HSV-1	dsDNA	Liver-cancer specific oncolytic virus (LCSOV).	Not mentioned	Hepatocellular Carcinoma (HCC)/Right flank	Intratumoral	LCSOV was very selective in the shrinking of HCC xenografts in mice	Fu et al. (2012)
HSV-1	dsDNA	A doubly fusogenic oHSV (Synco-2D)	CD8^+^ T-cells	Breast Cancer	Intratumoral	Synco-2D brought about the elimination of both primary and metastatic tumors.	Nakamori et al. (2004)
HSV-2	dsDNA	Deletion of the protein kinase domain of the viral ICP10 gene	Not mentioned	Metastatic Ovarian Cancer	Intraperitoneal	Obliteration of metastatic tumors in the peritoneal cavity of at least 87% of the mice	Fu et al. (2007)
HSV-1	dsDNA	EGF-PL-armed Synco-4 derived from Synco-2D	NK cells and Macrophages	Colon Tumor/right flank	Intratumoral	Incorporation of the chimeric molecules into oHSV improved the antitumor effect of the virotherapy.	Fu et al. (2020)
HSV-1	dsDNA	Ld0-GFP derived from oncolytic ICP0-null virus (d0-GFP)	Innate immune cells (specific ones not mentioned)	Hepatocellular carcinoma	Intratumoral and Intravenous	Increased survival of mice treated with Ld0-GFP	Luo et al. (2019)
HSV-1	dsDNA	oHSV-CD40L; murine CD40L engineered into oHSV	T cells and dendritic cells	Pancreatic ductal adenocarcinoma	Intratumoral	Repeated treatment with oHSV-CD40L increased the survival of mice and also offered them a long-term immunity from tumor relapse.	Wang et al. (2022)
HSV-1	dsDNA	G47△-mIL12: oHSV encoding a master anti-tumor cytokine, interleukin 12.	CD45^+^ leukocytes and CD8^+^ T cells	Triple-negative breast cancer/Breast	Intratumoral	G47△-mIL12 treatment inhibited the metastasis of cancer cells.	Ghouse et al. (2020)
HSV-2	dsDNA	ICP34.5 and ICP47 genes deleted	NK cells	Breast cancer	Intratumoral	Treatment slowed down the growth of tumor cells without causing weight loss of the mice	Zhao et al. (2014)
HSV-1	dsDNA	T-01 (deletion of the α47 and γ34.5 loci and replacement of the ICP6 gene with lacZ gene)	CD8^+^ T cells	Hepatocellular carcinoma	Intraperitoneal and Intravenous	T-01 treatment reduced tumor volumes in mice as compared to the control treatment	Nakatake et al. (2018)
HSV-1	dsDNA	G207 (deletion of γ34.5 genes and a lacZ insertion into the U_L_39 gene) and M002 (deletion of γ34.5 genes and expresses murine IL-12)	CD133+ and CD15^+^ cells	Pediatric Medulloblastoma	Intracerebral	Survival time of mice treated with G207 and M002 were significantly prolonged	Friedman et al. (2016)

**TABLE 2:** Summary of adenoviruses implicated as oncolytic viro-immunotherapeutic agents against cancer.

Oncolytic virus	Nucleic acid	Any genetic modification?	Immune Cells Involved	Cancer type/location	Delivery mode	Outcome	Reference
Ad5	dsDNA	E1B-19 deletion (*dl*250)	Immune system components not investigated	Pancreatic cancer	Intratumoral	Delayed tumor growth in mice treated with *dl*250 compared to the control-treated mice	Liu et al. (2004)
AdV	dsDNA	ICOVIR-15K-cBiTE [AdV engineered to express an EGFR-targeting bispecific T-cell-engager (BiTE)]	T cells	Not-specified	Intratumoral	Combined treatment of ICOVIR-15K-cBiTE with peripheral blood mononuclear cells increased the antitumor activity of ICOVIR-15K-cBiTE.	Fajardo et al. (2017)
AdV	dsDNA	ZD55-sflt-1 [*sflt*-1 (1–3) inserted into an E1B-55-kDa-deleted oncolytic adenovirus (ZD55)]	Not mentioned	Colorectal cancer	Intratumoral	50% survival in the ZD55-sflt-1 treated groups and 0% survival in the PBS (control) treated group.	Zhang et al. (2005)
Ad5	dsDNA	CV706 (E3-deleted)	Not mentioned	Prostate cancer	Intratumoral	Greater than 50% reduction of PSA in 5 patients treated with the highest dose levels of CV706	DeWeese et al. (2001)
Ad5	dsDNA	Ad5-△24-GMCSF [an oncolytic adenovirus coding for GMCSF (granulocyte macrophage colony-stimulating factor)]	CD8^+^ T cells	Different advanced metastatic cancers (hepatocellular, jejunum, breast, ovarian. gastric, medullar thyroid, mesothelioma, melanoma, colon, non-small cell lung, cervical, choroidal, ovarian, renal, leiomyosarcoma, and synovial)	Intratumoral or Intracavitary	Ad5-△24-GMCSF was discovered to be efficacious in 63% of the patients.	Cerullo et al. (2010)
Ad5	dsDNA	VCN-01; a derivative of ICOVIR-15K a cancer selective adenovirus	Not mentioned	Glioblastoma Multiforme/Brain	Intratumoral	A significant increase in the survival of mice treated with VCN-01 in two different mouse models for glioma	Vera et al. (2016)
Ad5	dsDNA	Ad5-△24RGD; contains a 24-base pair (24-bp) deletion in the CR2 of the E1A gene.	Not mentioned	Ovarian Cancer	Intraperitoneal	Mice treated with Ad5-△24RGD survived for more than 60 days and they did not show any evidence of intraperitoneal disease after treatment. Whereas the mice in the control group did not survive for up to 41 days and they developed tumors at the site of the injection.	Bauerschmitz et al. (2002)
Ad5	dsDNA	ZD55; deletion of E1B 55-kD gene. CD/5-FC is *Escherichia coli* prodrug-based therapy	Not mentioned	Colon Cancer	Intratumoral	At the end of the study 2 out of the six mice that were treated with ZD55-CD/5-FC were tumor free	Zhang et al. (2003)
Ad5	dsDNA	CG0070 encodes the cDNA for human GMCSF	Not mentioned	Bladder Cancer	Intratumoral	96% inhibition of tumor growth rate in the CG0070-treated mice as compared to the PBS-treated mice. Total tumor regression in half of the mice treated with CG0070.	Ramesh et al. (2006)
Ad5 and Ad3	dsDNA	Ad5/3-△24; a 24-bp deletion in CR2 of the E1A gene and an incorporation of the adenovirus serotype 3 knob in the Ad5 genome	Not mentioned	Ovarian Cancer	Intraperitoneal	There was no significant difference between the mice treated with a single injection or multiple injections of Ad5/3-△24. However, the overall survival was significantly better in mice treated with Ad5/3-△24 as compared to the control-treated.	Kanerva et al. (2003)
Ad5	dsDNA	Ad5-△24RGD; contains a 24-base pair (24-bp) deletion in the CR2 of the E1A gene.	Not mentioned	Cervical Cancer	Intratumoral or Intravenous	A significant reduction in the tumor size of mice treated with Ad5-△24RGD. The triple dose of Ad5-△24RGD produced a more pronounced effect as compared to the single dose.	Bauerschmitz et al. (2004)
Ad5	dsDNA	Ad5-△24-CpG; 18 immunostimulatory islands were engineered into the genome of Ad5-△24.	NK cells	Melanoma	Intratumoral	Ad5-△24-CpG significantly enhanced tumor control, in a murine model of melanoma, as compared to the controls.	Cerullo et al. (2012)
Ad5	dsDNA	Ad5/3-△24-GMCSF; a Ad5/3-△24 gene armed with human GMCSF.	CD8^+^ T-cells	Metastatic solid tumors	Intratumoral	Treatment with Ad5/3-△24-GMCSF resulted in disease control in 8 out of 12 patients	Koski et al. (2010)
Ad5	dsDNA	CG7870; this gene expresses E1a under control of the rat probasin promoter and E1B under control of the PSA promoter-enhancer. The Ad5 wild-type E3 region is also not deleted as seen in other Adv-based vectors.	Not mentioned	Prostate Cancer	Intratumoral	52 days after treatment, the average tumor volume for CG7870 treated and the radiation treated mice were 100% of baseline. Whereas that of the “CG7870 + radiation” treated group decrease to 20% of the baseline.	Dilley et al. (2005)
Ad5	dsDNA	*dl*309; a gene with E3 10.4/14.5, 14.7 kDa deletions.	Macrophages and CD8^+^ T-cells	No Specific cancer type. Cancer cells from four different carcinoma cell lines were used to develop four xenograft mouse models.	Intratumoral	*dl*309 was eliminated rapidly in four mouse models as compared to Ad5 and *dl*704. Macrophage infiltration due to *dl*309 treatment, and CD8^+^ T-cells infiltration due to Ad5 or *dl*704 treatment.	Wang et al. (2003)
		*dl*704; a gene with E3gp19 kDa deletion.					
		Ad5; E3 wild type adenovirus.					
Ad5	dsDNA	ZD55-IL-24; an insertion of an IL-24 expression cassette into the ZD55 gene	Not mentioned	Colorectal Cancer	Intratumoral	A significant suppression of tumor growth in mice treated with ZD55-IL-24 as compared to the saline-treated group	Zhao et al. (2005)
Ad5	dsDNA	Ad5-△24RGD; contains a 24-base pair (24-bp) deletion in the CR2 of the E1A gene.	CD8^+^ T-cell	Glioma	Intratumoral	Prolonged survival of glioma-bearing mice treated with Ad5-△24RGD	Jiang et al. (2014)
Ad5	dsDNA	VCN-01; It harbors a 24-base pair deletion in the E1A region. The E1A promoter has insertions of eight E2F-binding sites.	Not mentioned	Pediatric Osteosarcoma	Intravenous	VCN-01 showed a significant anti sarcoma effect in the metastatic osteosarcoma mouse model.	Martínez-Vélez et al. (2016)
Ad5	dsDNA	Ad-DHscIL12; Luciferase or IL-12 was incorporated into the E3 region of the adenovirus using a selective 6.7K/gp19K deletion.	The cells were generalized as leukocytes	Pancreatic Cancer	Intratumoral	Tumor growth reduction in mice treated with Ad-DHscIL12.	Bortolanza et al. (2009)
Ad5	dsDNA	Ad5-△24RGD; contains a 24-base pair (24-bp) deletion in the CR2 of the E1A gene.	CD4^+^ and CD8^+^ T-cells, and macrophages	Glioma	Intratumoral	Long-term survival in 50% of mice treated with Ad5-△24RGD.	Kleijn et al. (2014)

**TABLE 3:** Summary of poxviruses implicated as oncolytic viro-immunotherapeutic agents against cancer.

Oncolytic virus	Nucleic acid	Any genetic modification?	Immune Cells Involved	Cancer type/location	Delivery mode	Outcome	Reference
Oncolytic vaccinia virus (VV)	dsDNA	JX-594: is a thymidine kinase (TK) gene-inactivated oncolytic vaccinia virus expressing GM-CSF and lac-Z transgenes.	T-cells	Lung and Liver Cancer	Intravenous	JX-963 prevented the outgrowth of any noticeable lung or liver metastases in the mice.	Lee et al. (2010)
		JX-963; a TK and vaccinia growth factor deleted mutant expressing GM-CSF					
VV	dsDNA	vvDD-CXCL11; vaccina virus armed with the chemokine CXCL11	CD8^+^ T cells	Mesothelioma and Colon Cancer	Intraperitoneal	Much less tumor burden for vvDD-CXCL11-treated mice as compared to the controls	Liu et al. (2016)
VV	dsDNA	vvTRAIL; an oncolytic poxvirus expressing a membrane-bound TRAIL	Not mentioned	Colorectal Cancer	Intratumoral	Treatment with vvTRAIL did not have any significant effect on the mice. However, treating the mice with a combination of vvTRAIL and Oxaliplatin (Ox), increased the survival of the mice.	Ziauddin et al. (2010)
VV	dsDNA	CF33-GFP; a GFP-encoding chimeric virus with a *J2R* deletion.	CD8^+^ T cell	Lung Cancer	Intratumoral	CF33-GFP-treated mice had a longer survival duration compared to the PBS-treated mice.	Chaurasiya et al. (2020a)
VV	dsDNA	vvDD-CXCL11; vaccina virus armed with the chemokine CXCL11	T-cells	Colon and Ovarian Cancer	Intratumoral	Treatment with vvDD-CXCL11 alone or a combination of vvDD-CXCL11 and anti-PD-L1 antibody reduced the tumor burden of mice as compared to the PBS-treated.	Liu et al. (2017)
VV and Myxoma virus (vMyx)	dsDNA	vvDD-IL15Rα-YFP; vaccinia virus engineered to express the fusion protein IL15Rα-IL15 and the yellow fluorescent protein (YFP).	T cells and NK cells	Glioma	Intratumoral	A combination of both viruses resulted in the eradication of gliomas in most of the mice. Single treatment with vMyx-IL15Rα-tdTr was safe, but vvDD-IL15Rα-YFP caused ventriculitis-meningitis in mice	Tang et al. (2020)
		vMyx-IL15Rα-tdTr; Myxoma virus engineered to express the fusion protein IL15Rα-IL15 and tdTomato Red (tdTr).					
VV	dsDNA	CF33-hNIS-ΔF14.5; a chimeric virus that has its genes *J2R* and *F14.5L* deleted, and it also encodes the human sodium iodide symporter (hNIS) gene)	CD8^+^ T cell	Triple-Negative Breast Cancer	Intratumoral	A combinatory treatment with CF33-hNIS-ΔF14.5 and anti-PD-L1 antibody resulted in an absolute tumor regression in a triple-negative breast cancer mouse model. Whereas treatment with either CF33-hNIS-ΔF14.5 or anti-PD-L1 antibody did exert any significant therapeutic effect.	Chaurasiya et al. (2020b)
VV	dsDNA	WR-△4; a vaccina virus Western Reserve (WR) strain with deletions of four viral genes; *A48R*, *B18R*, *C11R*, and *J2R*.	Neutrophils	Melanoma	Intratumoral	Treatment with the wild type virus WR led to a small decrease in the tumor growth whereas treatment with WR-△4 resulted in a strong reduction in tumor expansion.	Mejías-Pérez et al. (2018)
VV	dsDNA	vA34R; a poxvirus engineered by the insertion of a mutated *A34R* gene into its viral backbone.	T cells	Peritoneal Carcinomatosis	Intraperitoneal	vA34R-treated mice showed a significant increase in the survival of the mice compared to the vvDDr (parent virus of vA34R) or PBS-treated mice.	Thirunavukarasu et al. (2013)
VV	dsDNA	vvDD-CXCL11; vaccina virus armed with the chemokine CXCL11	T cells and NK cells.	Colorectal Cancer	Intraperitoneal	A combinatory treatment with vvDD-CXCL11 and a chemokine modulating drug cocktail resulted in the most noteworthy antitumor activity.	Francis et al. (2016)

**TABLE 4:** Summary of paramyxoviruses implicated as oncolytic viro-immunotherapeutic agents against cancer.

Oncolytic virus	Nucleic acid	Any genetic modification?	Immune Cells Involved	Cancer type/location	Delivery mode	Outcome	Reference
Measles virus (MV); Edmonston B vaccine strain	Single-stranded, negative-sense, enveloped RNA	MV was encoded with Bispecific T-cell engagers (MV-BiTEs)	T cell	Melanoma, Adenocarcinom and Human colorectal carcinoma	Intraperitoneal, intratumoral and intravenous injections	Oncolytic efficacy was achieved against solid tumors	Speck et al. (2018)
Mumps virus (MuV); Urabe strain	Single-stranded, negative-sense RNA	Recombinant MuV-UCs were encoded with green fluorescence protein; (rMuVUC-GFP).	T cell	Human myeloma, Plasma cell leukemia,	Intravenous	The colon carcinoma and neuroblastoma cells had significant viral replication while most of the cell lines were not permissive to the MuV-UC mumps virus infection. MuV-UC viruses also had a significant infection in CT-26-LacZ mouse colon carcinoma cells and N2A mouse neuroblastoma cells *in vitro*	Ammayappan et al. (2016)
				Ovarian cancer, Lung adenocarcinoma, Hela-cervical cancer, Neuroblastoma, colon carcinoma, lung carcinoma, Plasmacytoma, breast cancer, Mesothelioma, Lymphoma, renal carcinoma,			
				Myeloma and glioma.			
Newcastle disease virus (NDV)	Single-stranded, negative-sense RNA	An attenuated NDV vaccine (from the Hertfordshire strain, MTH-68/H)	T cell	Glioblastoma multi-forme (GBM)	Intravenous	MTH-68/H, a live attenuated-oncolytic viral strain of NDV was shown to have significant antitumor activity against advanced high-grade glioma in four patients with bad prognoses. Oncolytic treatment with NDV increased survival rates up to 5–9 years with good quality patient lifestyles.	Csatary et al. (2004)
NDV; Italien strain	Single-stranded, negative-sense RNA	Recombinant NDV carrying intact cHAb18 gene (rNDV-18HL)	T cell	Cell lines were used; SMMC-7721, HepG2, HuH-7, and BHK-21	Intravenous	The rNDV-18HL-encoded cHAb18 antibody showed effective antitumor activity without affecting the virulence of NDV.	Wei et al. (2015)
NDV	Single-stranded, negative-sense RNA	An attenuated NDV vaccine from the PV7011 strain	T cell	Mostly human (colorectal, pancreatic, breast, non–small-cell lung) cancers	Intravenous	The first phase 1 dose escalation study for the PV7011 strain was reported.	Pecora et al. (2002)
						Patients had various levels of neutralizing antibodies with signs of tumor regressions after PV701 administration. Side effects include; headache, nausea, fever, hypotension dependent on dosing, etc. Positive results indicated possible prolonged anticancer therapy in patients with solid tumors.	
NDV	Single-stranded, negative-sense RNA	Novel recombinant rAPMV-4	T-cell	Melanoma cells, carcinoma cells, human melanoma, colon carcinoma.	Intratumoral	Novel recombinant rAPMV-4 was discovered to have greater antitumor properties than the clinical candidate; NDV.rAPMV-4 should further be translated into clinical trials as a major anticancer therapeutic for solid tumors.	Javaheri et al. (2022)
NDV	Single-stranded, negative-sense RNA	Three recombinant rNDV strains differing in IFN antagonism	T-cell	Fibrosarcoma, Aadenocarcinoma, monocytic leukemia, T cell lymphoblast-like, cervical cancer, hepatocarcinom, colon cancers, neuroblastoma, breast cancer	Intratumoral	Selective oncolysis can be improved by augmenting the innate immune responses of NDV.	Elankumaran et al. (2010)
Parainfluenza virus 5 (PIV5)	Single-stranded negative sense RNA	P/V gene (P/V-CPI_) mutation	T-cell	Human laryngeal cancer	Not mentioned	(P/V-CPI_) a mutated form of the PIV5 can destroy the majority of HEp-2 human laryngeal cancer cells but a certain population might emerge again over time indicating the need for combination therapy with chemotherapy	Fox and Parks, (2018)
Measles virus (MV)- Schwarz strain and mumps virus (MuV)- RIT 4385 strain	Single-stranded negative sense RNA	Attenuated measles (MV) and mumps (MuV) viruses	T-cell	Breast adenocarcinoma	Not mentioned	Unlike the previous opinion that tumor-associated macrophages (TAMs) have a negative impact on oncolytic virotherapy, this study showed that TAMs potentiate the anti-tumor ability of MeV and MuV. This contradiction could be because previous studies used murine tumor models in different oncolytic viruses while this study used *in vitro* models.	Tan et al. (2016)
Simian virus 5 (SV5).	Single-stranded negative sense RNA	G3A mutation was incorporated into the F protein of P/V-CPI-	Type I interferon (IFN)	Prostate cancer	Subcutaneous injection	PV simian virus 5 (SV5) with mutations in the P/V gene can selectively induce apoptosis in tumor cells and not affect normal cells. A virus expressing hyper-fusogenic glycoprotein retained IFN sensitivity and was more effective as a selective oncolytic vector.	Gainey et al. (2008)
NDV-73T	Single-stranded, negative-sense RNA	NA	T-cell	Melanoma	Subcutaneous	The stage II clinical trial was conducted with 83 patients whose melanoma cells were weekly injected with the virus. After 10 years of observation, over 60% of the patients were alive without recurrent disease. The outcome was an improvement of some historic controls indicating the role of NDV in stage II melanoma.	Cassel and Murray, (1992)
NDV- PV701	Single-stranded, negative-sense RNA	PV701; naturally attenuated strain of NDV	T-cell	Advanced-stage and incurable solid cancers	Intravenous	Phase 1 clinical trial with 16 patient’s enrollments. One patient had total remission while disease stabilization was observed in others.	Laurie et al. (2006)
MV-EZ	Single-stranded negative sense RNA	MV-EZ; an attenuated strain of MV	T-cell	T-cell lymphoma	Intravenous	Phase 1 clinical trial with 5 patients was done with various outcomes. One patient’s inoculated metastasis completely regressed while the other’s inoculated metastasis partially regressed.	Heinzerling et al. (2005)
MV; Urabe strain	Single-stranded negative sense RNA	NA	T-cell	Different cancer stages; 3 and 4	Local administration, peritumoral, intravenous, inhalation	Phase 1 clinical trial was done with 90 patients. Impressive or complete tumor regression was observed in 37 patients.	Asada, (1974)
MV; Urabe strain	Single-stranded negative sense RNA	An attenuated strain of MV; Urabe strain	T-cell	Uterine and ovarian cancer	Subcutaneous, intraperitoneal, intratumoral and intravenous	22 patients were recruited for the phase II clinical trial. Patients with large mass had an insignificant response to MV therapy while there was a significant clinical response in patients with malignant ascites or pleurites.	Shimizu et al. (1988)
Sendai Virus (SDV), Moscow strain	Single-stranded negative sense RNA	An attenuated strain of SDV; the Moscow virus	T-cell	Different cancer stages; 3 and 4	Intradermal, intratumoral	The treatment study was 47 individual cases. Various clinical responses were observed; 16 patients were not responsive to treatment, 6 patients had complete tumor regression and 25 patients had a partial response to treatment.	Matveeva et al. (2015)
NDV	Single-stranded negative sense RNA	NDV-infected mesenchymal stem cells (MSCs)	T-cell	Glioblastoma	Not mentioned	Dose-dependent cell death was induced by NDV in glioma cells and a low level of apoptosis in glioma stem cells. MCSc secreted factor(s) that increased the sensitization of the glioma cells to the oncolytic effect of NDV.	Kazimirsky et al. (2016)
NDV	Single-stranded negative sense RNA	Recombinant NDV LaSota L289A viruses	T-cell	Murine melanoma	Intraperitoneal	A murine melanoma model was used to evaluate the efficacy of IT injections of recombinant NDVs which indicated a significant antitumor effect.	Vijayakumar et al. (2020)
MTH-68/H-NDV	Single-stranded negative sense RNA	Attenuated strain; MTH-68/H-NDV	T-cell	Anaplastic astrocytoma	Intavenous, inhalation	A 12-year-old patient with anaplastic astrocytoma had both NDV and valproic acid (VPA) treatment which led to significant involution of the thalamus gland. A new tumor developed which was resistant to the treatment.	Wagner et al. (2006)
Canine distemper virus (CDV), MV	Single-stranded negative sense RNA	Attenuated CDV expressing enhanced green fluorescent protein	T-cell, B-cell	Canine lymphoid cancer, canine osteosarcoma, canine melanoma and marmoset B95a lymphoblastoid cancer	Intratumoral, intravenous	Attenuated CDV can be a useful source of treatment for canine lymphoma. This treatment could be applicable to the treatment of human non-Hodgkin’s lymphoma soon.	Suter et al. (2005)

## 2 The principle of oncolytic viro-immunotherapy in cancer treatment

**FIGURE 1:**
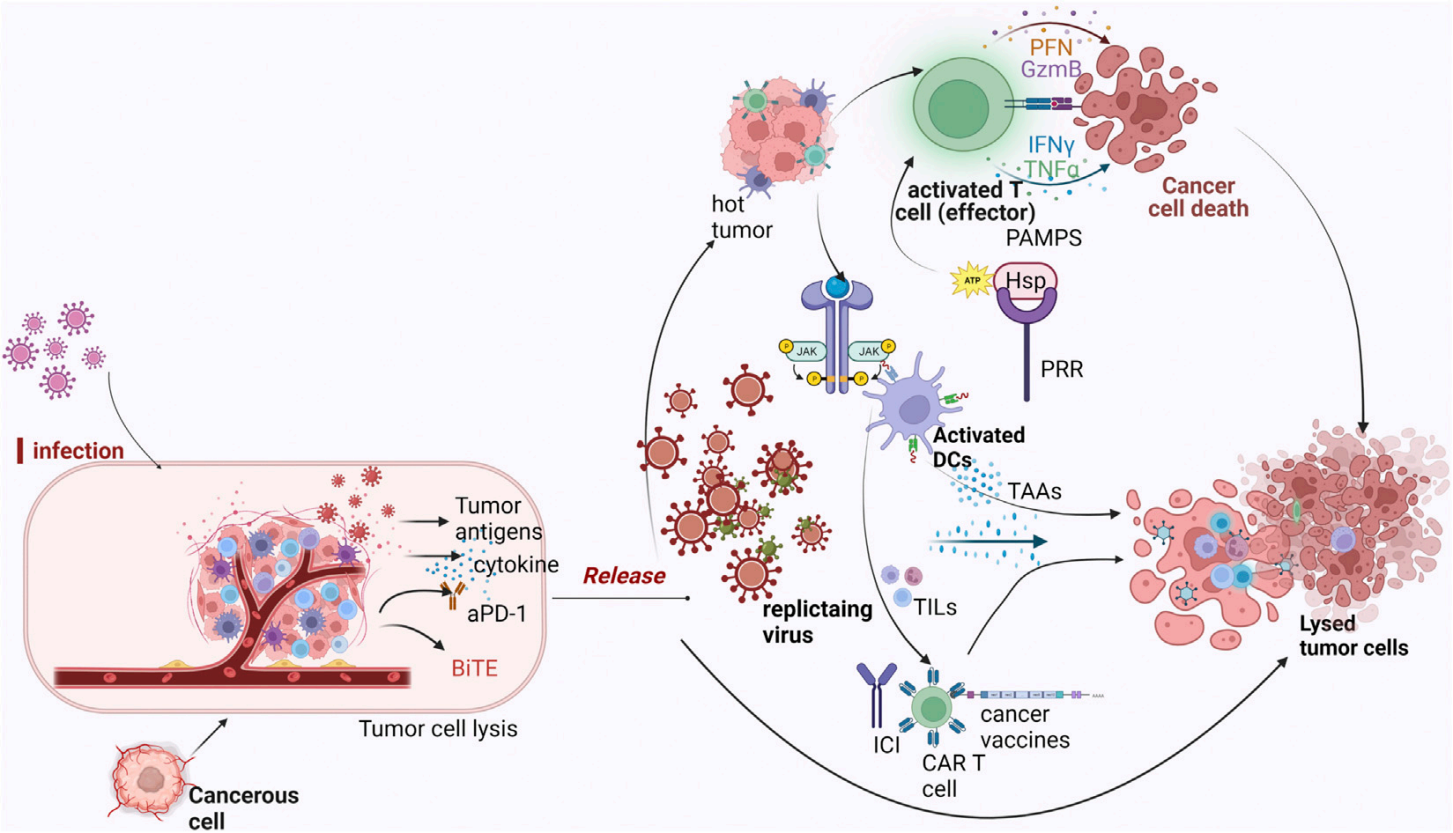
Mechanism of oncolytic viruses as immunotherapeutic agents against tumor cells. Legend: Oncolytic viruses infect tumor cells and recondition the tumor microenvironment for the effector cells to be activated. Once they start replicating within the tumor, direct tumor lysis occurs which causes the release of DAMPS and PAMPS which are recognized by PRR expressed on immune subsets like DCs, NK cells and so on. As a result of this interaction, inflammatory cytokines are recruited, hence, attracting other immune cells. Replication of the virus also initiates the expression of TAA which are captured by DCs for presentation to T cells and traffics T cells to the tumor causing ICD. Oncolytic viruses when combined with any of CAR-T therapy, checkpoint inhibitors or cancer vaccines further ensure favorable response, with the presence of TAA and TIL’s enhancing their effects.

Oncolytic viruses can preferentially infect and destroy tumor cells while stimulating and engaging the immune system through different mechanisms which include: modulation of the TME (that is, converting cold tumors to hot), directly lysing tumor cells or combining therapeutically with cancer immunotherapies. This principle is summarized by the illustrations in Figure 1.

### 2.1 Reconditioning/modulating the TME (converting cold tumors to hot)

The immunological phenotype and landscape of the TME is an important factor in determining disease prognosis and therapeutic efficacy (Achard et al., 2018). Cold tumors are characterized by the inability of effector cells to infiltrate the tumor. Oncolytic viruses make cold tumors hot by taking advantage of cancer’s telltale signs, that is, hallmarks of cancer (Engeland, 2020). Some of these cancer telltale signs include invasion and metastasis, sustained proliferation, induction of angiogenesis, resistance to apoptosis, and evasion of immune surveillance and growth suppressors. These attributes make the tumor microenvironment a dynamic and complex one comprising of not just individual malignant cells but also vascular endothelial cells, fibroblasts, tumor-resident or migratory immune cells, stroma and vasculature; all of which contribute to their immunosuppressive capabilities (Gujar et al., 2018). Reciprocal crosstalk between the tumor and stroma further promotes their invasiveness and metastasis. Attempts by the host to initiate an immune response against the tumor lead to an oppositional response by recruiting immunosuppressive cells to the tumor milieu, hence building an impenetrable fortress (Achard et al., 2018). They stop the infiltration of T cells to the tumor site making the tumor microenvironment cold. Since most current immunotherapies involve harnessing the immune T cell responses to fight cancer, it is only logical to introduce factors that would stimulate their activity. Introducing OVs to the tumor milieu helps reshape the tumor milieu by inducing an acute viral infection that could potentially stimulate inflammation and immune cell infiltration to the tumor site (Samson et al., 2018). For example, oncolytic vaccinia and vesicular stomatitis virus (VSV) can cut off tumor blood supply, enhance T cell infiltration and consequently inhibit tumor progression by targeting the tumor vasculature (Shi et al., 2020). Achard et al. (2018) describe this as the ability of OV to wake up the tumors from an immunological coma. T cell infiltration into the tumor bed makes the environment hot and appropriate for other immunotherapies to function. Once OVs successfully enter the tumor bed, they initiate immunogenic cell death by direct lysis of these OV-infected tumor cells. They can reverse the immunosuppressive environment in the tumor milieu and enable recognition of tumor associated antigens (TAA) by the T cells. This process awakens the immune system within the TME and is just the first step that results in a prolonged antitumor immune response. For example, studies have shown that vesicular stomatitis virus or reovirus primes the adaptive immune response eliciting T cell mediated immunity with evident signs of tumor regression (Diaz et al., 2007).

### 2.2 Direct lysis of tumor cells

A limitation of cancer cells is their flawed antiviral response pathways, and this defect makes them even more susceptible to OVs. Some of these signaling pathways involved in viral clearance include toll-like receptor (TLR), Janus kinase-signal transducer and activator of transcription (JAK-STAT), protein kinase RNA-activated (PKR) pathways, which are either absent or inhibited in cancer cells; this is exploited by OVs to recondition the tumor milieu (Guo et al., 2017). Direct lysis of OV-infected tumor causes the release of pathogen-associated molecular patterns (PAMPS) such as viral nucleic acids and proteins, and damage-associated molecular patterns (DAMPS) such as heat-shock proteins (HSP), adenosine triphosphate (ATP), ecto-calreticulin, High mobility group box protein 1 (HMGB1) (Lichty et al., 2014). These PAMPS are recognized by pattern-recognition receptors (PRR) expressed on various immune subsets like natural killer (NK) Cells, dendritic cells (DCs) and macrophages within the tumor bed (Diaz et al., 2007). With this recognition, inflammatory cytokines like IFN-α, IFN-γ, IL-12, IL-6 are produced which leads to the recruitment of other immune cells from peripheral organs thereby eliciting anti-viral and anti-tumor immune responses leading to Immunogenic cell death (ICD) (Diaz et al., 2007; Kroemer et al., 2013). For example, the oncolytic MeV (a reovirus with dsRNA genome) activates DCs *via* PKR signaling, induces Toll-like receptors (TLR) and/or RIG-I-like receptors (RLR) and secrets some of these pro-inflammatory cytokines like IFN-γ to induce anti-tumor immunity (Errington et al., 2008; Achard et al., 2017). In addition, tumor associated antigens (TAA), or tumor-specific antigens (TSA) are released within the tumor as a result of selective replication and tumor lysis, serving as adjuvants for adaptive immunity. Once at the site, DCs capture these TAA for cross-presentation to T cells. This leads to the priming cum activation, proliferation and trafficking of antigen-specific polyclonal T cells (Errington et al., 2008). These activated antigen specific CD8^+^ and CD4^+^ T cells through their cytotoxic effect causes immunogenic cell death in the tumor cells. In numerous preclinical experiments, this mechanism has been verified (Shi et al., 2020). Release of OV into the tumor can also induce *in situ* vaccination as a result of their spread and uptake by DCs (Lichty et al., 2014). This *in situ* vaccination is a result of the induction of specific TAA-specific adaptive immune responses and is an important component of the success of OV immunotherapy. An example is the T-VEC, an oncolytic HSV-1 which was licensed by the FDA for the treatment of metastatic melanoma (Bastin et al., 2016). It has deletions in the genes ICP34.5, ICP47 and expresses GM-CSF (Granulocyte-macrophage colony-stimulating factor) (Guo et al., 2017). Loss in ICP34.5 and ICP47 confers selectivity to cancer cells and enhances antigen presentation respectively, while GM-CSF aids APC maturation, activation and in turn trafficking of immune cells and induction of systemic antitumor immunity (Kaufman et al., 2013; Guo et al., 2017).

### 2.3 Combination therapy with cancer immunotherapies

**FIGURE 2:**
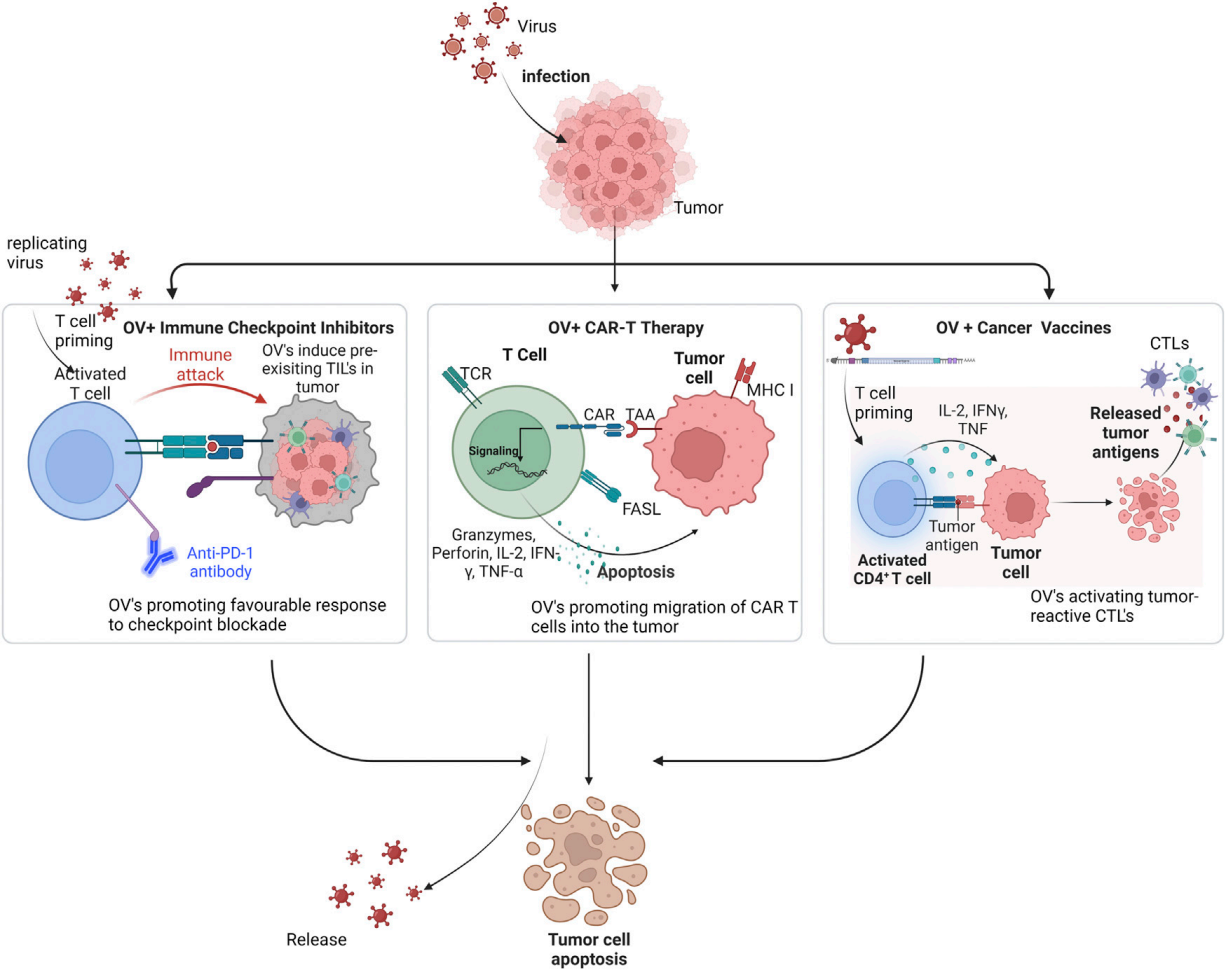
Mechanism of oncolytic viruses in combination with other immunotherapies against tumor cells. Legend: Incorporating OV’s with Checkpoint inhibitors primes T cells and makes tumor-infiltrating lymphocytes (TILs) readily available, thereby increasing response rate to this therapy. With CAR T therapy, OV’s deliver cytokines and chemokines to drive migration of CAR T cells into the tumor, thereby, synergistically inducing viral infection and immunogenic cell death. In cancer vaccines, OV’s recruit cytokines that activate tumor-reactive CTLs like the T helper cells *via* the released tumor antigens.

Dwelling on the mechanisms explained above, OVs are suited for combinational therapy approaches to enhance anti-tumor immunity. Therefore, OVs are being explored as antigen-agonists, increasing the action of checkpoint inhibitors, or adoptive cell treatments and cancer vaccines due to their significant potential to combat cancer (Cockle et al., 2016). They have the leverage of being ideal agents that may be used in synergy with available cancer immunotherapies to further augment positive treatment effects in several cancer types (Figure 2).

#### 2.3.1 Oncolytic viruses with checkpoint inhibitors

Checkpoint inhibitors prevent checkpoint proteins from binding/interacting with their partner proteins with the purpose of interrupting immunosuppression by tumor signals. T cells express checkpoint proteins like CLA4, PD-1, which help keep the immune system in check while cancer cells express PD-L1, PDL-2, VISTA (Pardoll, 2012; Topalian et al., 2014). Cancer cells unfortunately hijack these mechanisms to hinder anti-tumor immunity. There are antibodies that have been developed to target these checkpoint inhibitors; pembrolizumab and nivolumab targeting PD-1, ipilimumab targeting CTLA-4 (Patnaik et al., 2015). These monoclonal antibodies have shown encouraging results in several solid tumors (Wolchok et al., 2017). However, a common shortcoming is the low response rate due to minimal levels of tumor-infiltrating lymphocytes (TILs) (Jacquelot et al., 2017). Pre-clinical and clinical studies have emphasized the importance of pre-existing TILs in the TME because it promotes favorable response to checkpoint blockade (Wolchok et al., 2017). Incorporating OV has the prospects of overcoming these deficiencies. In numerous clinical trials, the interaction of OVs with immunological checkpoints is being studied, with PD-1/PD-L1 and CTLA-4 combinations making the most progress. For example, Samson et al. (2018), in a pre-clinical study published in 2018 reported a significant increase in cytotoxic CD8^+^ T cell infiltration in TNBC mouse model after treatment with oncolytic Maraba rhabdovirus and reovirus in combination with PD-1 inhibitors. An increase in response rate of about 33% was reported in advanced melanoma after combinational treatment with TVEC and ipilimumab in a phase I clinical trial when compared with either treatment alone (Ribas et al., 2017; Samson et al., 2018). Other examples of current studies in clinical trials include Phase I clinical trial of oncolytic virus injection RT-01 and PD-1 combination therapy (NCT05228119); Phase II clinical trials of Pexa-vec oncolytic virus (vaccinia virus) in combination with Tremelimunab (binds CTLA-4) or Durvalumab (binds PD-L1) in patients with refractory metastatic colorectal cancer (NCT03206073). Other studies have also shown that OVs can effectively act as neoadjuvant to prime the tumor microenvironment for immune checkpoint therapy (Bourgeois-Daigneault et al., 2018). While promising results are seen with this combinatorial therapy, a caveat is the possibility of abrogating OV replication and tumor infection due to excessive priming of systemic antiviral responses (Shi et al., 2020).

#### 2.3.2 Oncolytic viruses with chimeric antigen receptor T cell therapy

Adoptive therapy is hinged on the premise of *ex vivo* expansion of the patients’ T cells and reinfusion of these expanded, tumor-reactive T cells. In CAR-T cell therapy, the T cells can recognize tumor antigens through the CAR structure on T cells. These CAR-T cells infiltrate the tumor cells and eliminate them based on their antigens (Houot et al., 2015), with the ability to recognize TAA independently of MHC being their strong point. So far, they have shown promising effects and have been approved by FDA for the treatment of B-cell malignancies (Lichtman and Dotti, 2017). However, they have shown limited progress in solid tumors owing to the restricted trafficking of T cells into the tumor (Sauter et al., 2019). The ability of oncolytic viruses to induce viral infection and immunogenic cell death can be exploited in synergy with CAR-T cell therapy (Ajina and Maher, 2017). The possibility of engineering oncolytic viruses to deliver T cell chemokines and cytokinesis is increasingly gaining recognition and can be used to promote stimulation and migration of CAR-T cells into the tumor. For example, an engineered vaccinia virus that produces CXCL11 can increase T cell trafficking into a subcutaneous tumor. This enhanced recruitment of antigen-specific T cells after CAR-T cell administration significantly improved anti-tumor immunity (Moon et al., 2018). OVs can also be engineered with EGFR-targeting bispecific T cell engagers (BiTE) or re-wired to produce antibodies against checkpoint inhibitors to enhance CAR-T cell therapy. BiTEs are fusion proteins and they guide polyclonal T lymphocytes towards tumor cells without the aid of MHC. Because of this, they are able to elicit anti-tumor responses at low dosages (Heidbuechel and Engeland, 2021). They have shown promising results in the treatment of hematological malignancies (Viardot et al., 2016). A study by Wing et al. (2018) reported that oncolytic adenovirus engineered with EGFR-targeting BiTE improved the activation and proliferation of CAR-T cells, improving survival in the mouse model. In another study, some researchers generated an oncolytic virus that expresses TNFα- and IL-2 (Ad-mTNFα-mIL2) and in combination with CAR-T cell therapy, they were able to treat human pancreatic adenocarcinoma-xenograft immunodeficient mice. They discovered that the combination therapy enhanced T cell trafficking into the immunosuppressive milieu leading to DC maturation and M1 macrophage polarization (Watanabe et al., 2018).

#### 2.3.3 Oncolytic viruses with cancer vaccines

Cancer vaccines are designed with the intent to induce immune responses (cellular and humoral) *in vivo* for immunological memory to prevent and control tumor growth. Cancer vaccines include peptide, DNA, RNA and DC vaccines, all of which are in clinical trials for treatment of different solid malignancies like melanoma, glioma and colon carcinoma. A major drawback to this treatment option is their low recruitment profile of T helper cells and major histocompatibility complex II (MHC II) epitope on the surface of DC leading to less efficiency of the T cell anti-tumor effects. Thus, combining OVs with cancer vaccines has the potential to activate tumor reactive cytotoxic T lymphocytes (CTLs) (Li et al., 2017). The potential of this combinatorial therapy in booting priming of T cell responses has been reported. An example is the chemokine CCR5-expressing oncolytic vaccinia virus vvCCR5 which when mixed with DC1 (type-1-polarized DCs) triggered chemo taxis of lymphocytes *in vitro* and *in vivo* (Li et al., 2011). For the most part, most of the OV-cancer vaccines target the TAAs and are very promising; they can utilize the mechanism of turning the cold tumors into hot in order to elicit anti T cell responses (Shi et al., 2020).

## 3 Different oncolytic viruses applied as Immunotherapeutic agents in cancer treatment

Various oncolytic viruses have been exploited to treat different forms of cancers which include; herpesviruses, adenoviruses, poxviruses, rhabdoviruses, paramyxoviruses and reoviruses. The activities of some of these types of viruses in the fight against different forms of cancer both at the experimental and clinical stages are discussed below:

### 3.1 Herpesviruses

Herpesviruses are DNA viruses which are capable of establishing lytic and latent modes of infection in their hosts. Among herpesviruses, the utility of herpes simplex virus-1 (HSV-1) as an oncolytic agent has been explored the most (Koch et al., 2020; Saravanan et al., 2022). The large genome capacity of HSV-1 allows its use as a vector for delivering choice transgenes to cancer cells (Burton et al., 2002; Saravanan et al., 2022). Talimogene laherparepvec (T-VEC) which is derived from JS1, a primary isolate of HSV-1, was the first oncolytic virus to be approved for public use in the US for the treatment of advanced melanoma (Pol et al., 2016).

**FIGURE 3:**
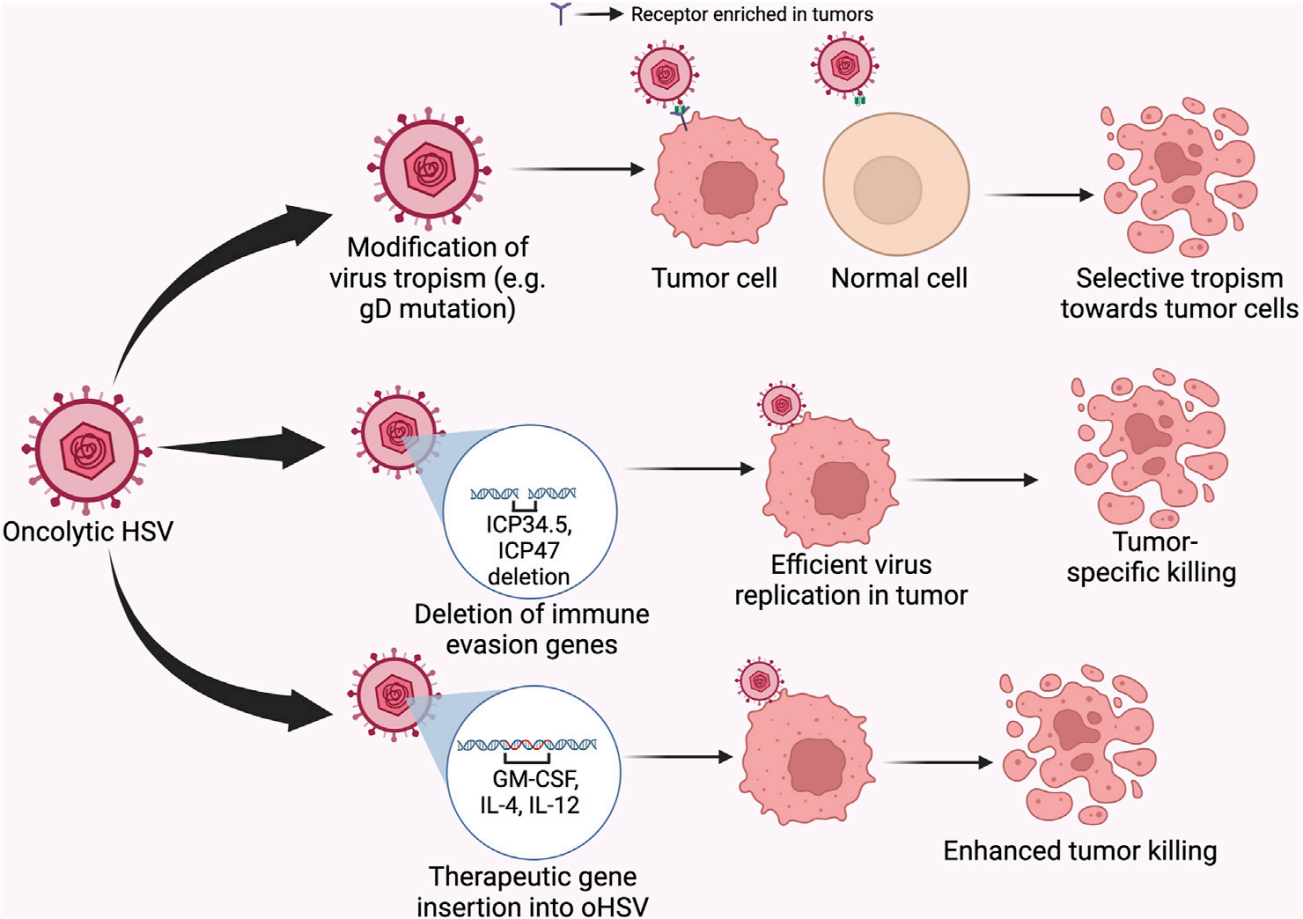
Different mechanisms by which oncolytic HSV achieve tumor killing. Legend: Modifications within the viral glycoprotein D to redirect tropism or to other regions of the genome to alter the interaction of the virus with intrinsic antiviral pathways in the cell, or the introduction of therapeutic genes within the genome of OVs provide mechanisms for achieving or improving targeted-killing of cancer cells.

The selectivity of oncolytic HSV (oHSV) for cancer cells is achieved through viral tropism and replicative fitness. The deletion of ICP34.5—a leaky late gene of HSV-1 in T-VEC relieves protein kinase R (PKR)-induced block to cellular protein synthesis, which is caused by the inactivation of the eukaryotic translation initiation factor, eIF2α. Since cancer cells often have a dysfunctional PKR, T-VEC selectively kills tumor cells but not normal cells as shown in Figure 3 (Liu et al., 2003; Saravanan et al., 2022). In addition, the deletion of ICP47 and the enhanced expression of US11 in T-VEC were shown to promote tumor clearance (Liu et al., 2003). The defectivity in the cyclic GMP–AMP synthase (cGAS)–stimulator of interferon genes (STING) signaling pathway in some cancer cells also provides an Achille’s heel for enhancing viral oncolysis (Xia et al., 2016; de Queiroz et al., 2019).

HSV-1 entry is facilitated by the interaction of the viral glycoprotein D (gD) with different cellular receptors, including nectin-1, herpes virus entry mediator and 3-O sulfated heparan sulfate (Shibata et al., 2016). Since some tumors have decreased expression of nectin-1, to enhance infectivity as well as selectivity, the residues on gD necessary for interaction with nectin-1 were replaced with a single chain antibody targeting epithelial cell adhesion activating molecule (EpCAM), thereby detargeting the virus from nectin-1 and retargeting virus tropism towards cells with surface expression of EpCAM (Shibata et al., 2016). Of note, EpCAM has long been identified as a prognostic factor for many human cancers (Baeuerle and Gires, 2007). Similar mutations have been used to direct virus tropism towards tumor cells expressing HER2 and EGFR to achieve oncolysis (Menotti et al., 2006; Uchida et al., 2013). Loss of the virus-encoded thymidine kinase (TK) gene also allows for tumor-specific virus replication (Martuza et al., 1991), but this mutation might hinder responsiveness to acyclovir (the replication of HSV-1 is naturally suppressed by acyclovir) under potential situations of uncontrolled virus replication or replication in unwanted sites (Gilbert et al., 2002).

To enhance the immunological response against tumors, T-VEC was engineered to express granulocyte-macrophage colony-stimulating factor (GM-CSF), thereby promoting the recruitment of T cells to the site of the tumor (Liu et al., 2003). Since ICP47 normally prevents antigen presentation, the loss of ICP47 in T-VEC proved a useful mutation in suppressing tumor growth. Among several mechanisms for immune escape, some tumors express programmed death ligand 1 (PD-L1), thereby inhibiting the activation of T cells infiltrating the tumor microenvironment (TME) (Jiang et al., 2019). Co-administration of T-VEC with anti-PD-1 antibody, or engineering oHSV to express a single chain antibody against PD-1 (programmed cell death protein 1) was shown to suppress tumor growth (Ribas et al., 2017; Passaro et al., 2019).

Interleukin-4 (IL-4)-expressing oHSV promotes tumor clearance, whereas, the expression of IL-10 suppresses survival of the mice bearing the tumor, lending credence to the importance of the nature of the immune response at the site of the tumor in promoting or preventing tumor clearance (Andreansky et al., 1998). In another study, encoding IL-12, a cytokine that promotes the killing ability of natural killer cells and cytotoxic T cells within the genome of oHSV similarly improves the cytolytic activity of the immune cells (Parker et al., 2000). Besides the primary immune response elicited following treatment with oHSV, primary infection with oHSV was also shown to prevent against subsequent exposure to the tumor (Liu et al., 2003). Different studies that have applied herpesviruses as immunotherapeutic agents in the fight against cancer are presented in Table 1.

In addition to T-VEC, other oHSVs have been objects of clinical trials including G207, 1716, HF10, and ND1020 (Koch et al., 2020). HSV-1716 (Seprehvir) was the first oHSV to be administered *via* intravenous route in humans (Streby et al., 2019). Intravenous delivery of Seprehvir was well tolerated, but the virus was not recovered from the tumor. Since the patients recruited for this study also received other therapies besides Seprehvir, it was difficult to determine the contribution of Seprehvir alone in the survival of the patients (Streby et al., 2019). This study indicates a need for more research in understanding the factors contributing to efficient biodistribution of oHSV, as this would prove useful in targeting metastatic tumors.

Engineering oHSVs to express reporter genes allows easy tracking of the virus within animal hosts and provides a mechanism for detecting the virus in case of spread to sites outside the tumor (Mineta et al., 1995; Peters and Rabkin, 2015). This consideration becomes especially important given reports of possibilities for virus replication in non-tumor sites in mice after receiving oHSVs (Kesari et al., 1998). In the future, combining the beneficial mutations seen in different oHSVs with the transgenic delivery of therapeutic genes in single oHSV vectors may further improve the efficacy of oHSVs against human tumors. For example, designing single oHSVs with tumor-specific tropism, selective replication in tumors, and improved ability to promote tumor infiltration and sustained anti-tumor activities of immune cells at the TME would significantly improve the oncolytic potential of oHSVs against several human cancers.

### 3.2 Adenoviruses

Adenoviruses (AdVs) are small, non-enveloped DNA viruses with genome size of ∼36 kb. The viral genome is divided into early and late transcription units. The genome is enclosed by an icosahedral capsid with penton borne fibers projecting from each of the 12 vertices of the icosahedron. The fiber knob at the terminus of the fiber interacts with cellular receptors to dictate the tropism of AdV types (Barnett et al., 2002). Secondary interactions between the penton base proteins and integrin are also important for virus internalization (Stepanenko and Chekhonin, 2018). AdV type 5 (Ad5) is the most common AdV utilized for oncolytic virotherapy as evident from the findings in Table 2. The cellular receptor for Ad5, coxsackie virus and adenovirus receptor (CAR), is expressed at low levels in some tumors (Reeh et al., 2013), thereby reducing the tropism of the virus for cancer cells. However, generating a chimera of AdV types carrying knobs derived from other AdV types on the background of Ad5 allows for enhanced transduction and selectivity of oncolytic AdVs (oAdVs) into tumors (Koodie et al., 2019; Gao et al., 2020; Zafar et al., 2021). Other mechanisms for enhancing tropism of oAdVs are reviewed here (Stepanenko and Chekhonin, 2018). Interestingly, the loss of the CAR and/or integrin binding was shown to promote the hematogenous distribution of oAdV, suggesting that this mechanism may be exploited for targeting metastatic tumors (Akiyama et al., 2004).

The early gene products, E1A and E1B target the retinoblastoma protein (pRb) and p53 respectively to enforce S phase entry and virus replication (Tessier et al., 2021). An E1B-55 kDa-deficient AdV mutant (ONYX-015) was shown to replicate in, and lyse only p53-deficient cells, thereby providing selectivity between tumors and normal cells (Bischoff et al., 1996). Subsequent studies revealed that the mechanism driving the selectivity of ONYX-015 might be more complex than was previously thought (Kirn, 2001; Edwards et al., 2002). ONYX-015 became the first engineered oncolytic virus to be administered to humans, demonstrating safety at high doses independent of the route of administration in clinical trials (Kirn, 2001). Nevertheless, ONYX-015 by itself failed to promote tumor regression, but showed promise in combinational approaches with chemotherapy (Kirn, 2001; Garber, 2006). Others have also shown that tumor selectivity can also be achieved by the deletion of E1B-19 kDa (Liu et al., 2004). Another oAdV, H101, is similar to ONYX-015 except it carries a mutation in the E3 gene region which is associated with immune evasion (Zhang et al., 2021). H101 was well tolerated and demonstrated anti-tumoral activity in patients (Lu et al., 2004). It was eventually approved for human use in China in 2005 (Garber, 2006).

**FIGURE 4:**
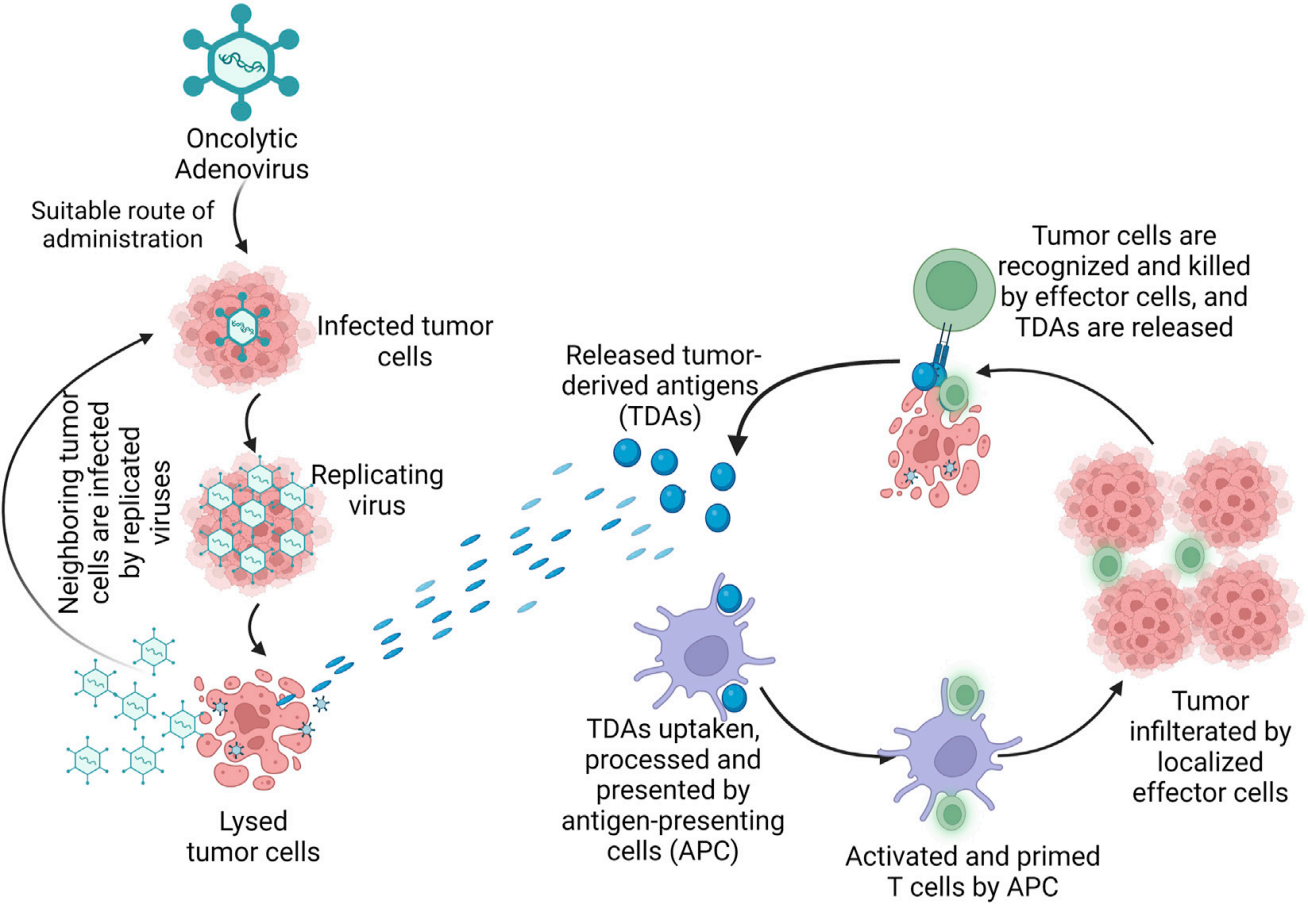
Mechanism of oncolytic adenovirus in cancer immunotherapy. Legend: The oncolytic adenovirus once administered by suitable route, infects the tumor cells and replicates in the tumor, causing direct lysis of the tumor. Once the tumor is lysed, tumor-derived antigens (TDAs) are released and the cells of the immune system are activated. The TDAs are uptaken, processed and presented by antigen-presenting cells (APC); the APC activates and primes the T cells which result in tumor-specific killing by the effector cells of the host immune system.

Another oAdV, ZD55-sflt-1 which was constructed on the ONYX-015 backbone, expressed an anti-angiogenic factor and further decreased survival of tumor cells when compared to ONYX-015 (Zhang et al., 2005). Engineering oAdv to encode vehicle genes under the regulation of tumor-specific promoters provided another means for achieving tumor selectivity (Abudoureyimu et al., 2019). The safety profile of ONYX-015 and the efficacy of H101 even at high doses prove the suitability of oAdVs as viro-immunotherapeutic agents and support the need for further investigations to improve their efficacy against different human tumors. The mechanism of cancer immunotherapy by adenoviruses is presented in Figure 4.

### 3.3 Poxviruses

Poxviruses (POXVs) are large DNA viruses with linear, double stranded genome. They replicate entirely in the cytoplasm of infected cells in structures called viral factories (Schramm and Locker, 2005). POXVs package within their particles, RNA polymerases as well as transcription factors required for early gene expression. The virus encodes its own RNA and DNA polymerases as well as other factors required for viral gene expression and replication in the cytoplasm (Wittek, 1982). The genome comprises of a central conserved region and terminal regions that are variable among POXVs, and which encodes factors for evading the host immune response (Wittek, 1982). Variola virus is the etiologic agent for smallpox in humans. A related POXV, vaccinia virus (VV) was used in its live, attenuated form as the vaccine for smallpox in humans.

The fast replication kinetics of VV, their efficient mechanism for cell-to-cell spread, their suitability for intravenous dissemination, and their long history of use as a vaccine agent make them ideal candidates for oncolytic virotherapy (Doceul et al., 2010; Irwin and Evans, 2012). Moreover, the large genome of POXVs generally allows them to serve as vectors for the expression of therapeutic genes. VV is the most common POXV applied in viral oncolysis. VV encodes a TK gene whose loss is associated with decreased virulence (Buller et al., 1985). Unlike normal cells, cancer cells produce abnormally high levels of a human homolog, TK1 (Bitter et al., 2020). Hence, the deletion of TK in oncolytic vaccinia virus (oVV) is often used to achieve selectivity for cancer cells. Different oncolytic poxviruses that have been used in the treatment of various cancer types are presented in Table 3.

**FIGURE 5:**
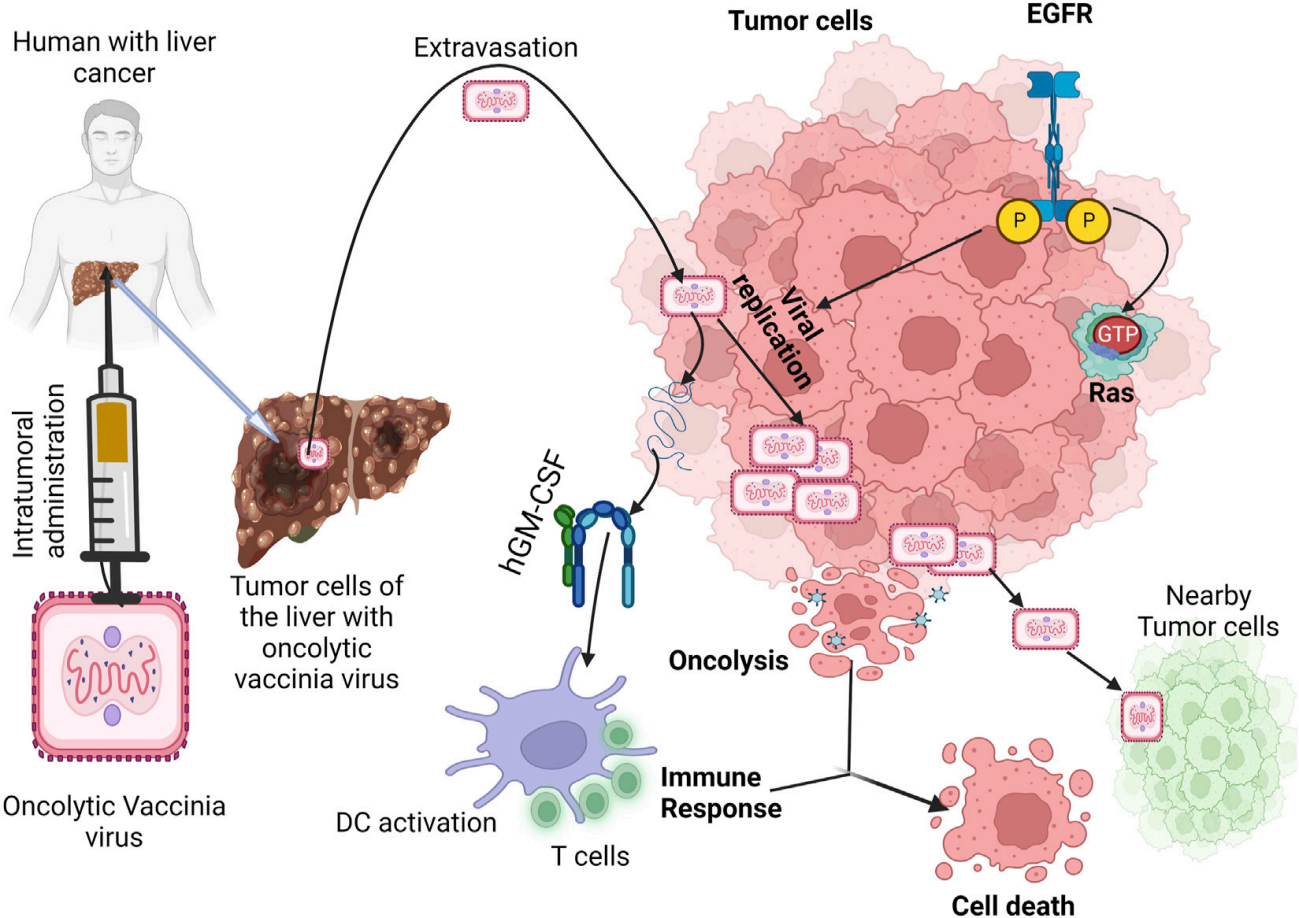
Mechanism of oncolytic vaccinia virus in cancer immunotherapy. Legend: The oncolytic vaccinia virus replicates in the tumor cell *via* active pathway of EGFR-RAS. Once the replication process is completed, tumor cell death occurs by viral-induced oncolysis and GM-CSF is expressed simultaneously to activate immune-induced cell death (Hernandez-Gea et al., 2013).

One of the oVVs that has made it to clinical trials is JX-594 (Kim et al., 2006). JX-594 is gene-inactivated for TK and is also engineered to encode GM-CSF (Kim et al., 2006). The illustration showing the mechanism of action of oncolytic vaccinia virus is shown in Figure 5. Phase I application of JX-594 *via* intratumoral administration against liver and skin cancers demonstrated safety and anti-tumor activity with hyperbilirubinemia as a dose-limiting symptom (Mastrangelo et al., 1999; Park et al., 2008). Other POXVs that have been investigated as oncolytic agents include GLC-1h68, VV-FCU1, JX-795, JX-963, and vvDD which are all defective for TK expression, in addition to other unique mutations or insertions (McCart et al., 2001; Kirn et al., 2007; Thorne et al., 2007; Foloppe et al., 2008; Zhang et al., 2009). Expressing HSV-derived, truncated TK in oVV renders the virus susceptible to treatment with ganciclovir without affecting tumor selectivity, thereby strengthening the safety profile of oVV (Islam et al., 2020). oVVs deleted for virus-encoded immune evasion genes also hold potential as agents for driving tumoral selectivity in future studies, leaving intact the viral TK gene (Ho et al., 2021).

As one of the mechanisms to dampen the immune response at the TME, cancer cells in solid tumors outcompete lymphocytes for nutrients, resulting in metabolic insufficiency (Siska and Rathmell, 2015; Rivadeneira et al., 2019). Leptin-encoding oVV overcomes this effect, increasing T cell activity and memory as well as promoting tumor regression in mice (Rivadeneira et al., 2019). Cytokines secreted by stromal cells in the TME are known to participate in promoting tumor initiation and progression (Pearl et al., 2019). Consequently, reprogramming the TME through vvDD-IL-12-FG-mediated local delivery of IL-12 to the tumor promoted elevated the IFN-γ levels, increased the infiltration of CD8^+^ T cells, decreased the T cell exhaustion; consequently resulting in increased tumor clearance (Ge et al., 2020). Of note, the combination of vvDD-IL-12-FG with a PD-1 inhibitor dramatically enhanced the survival of mice bearing advanced tumors. It would be interesting to investigate the outcome of this combinational therapy in clinical trials (Ge et al., 2020).

### 3.4 Paramyxoviruses

**FIGURE 6:**
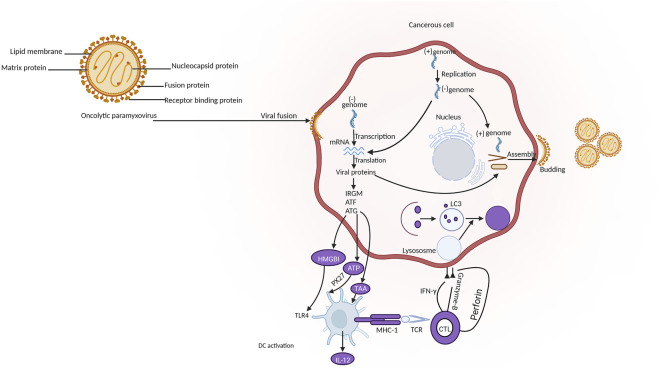
Mechanism of oncolytic paramyxovirus in cancer immunotherapy. Legend: The pathway involves the release of tumor-associated antigen (TAA) and damage-associated molecular patterns (DAMPs) by the tumor cells after the replication of oncolytic paramyxovirus. This is followed by the recognition by pattern recognition receptors (PRRs) on antigen presenting cells (APCs) and the release of inflammatory cytokines. TAAs can also be introduced by mature APCs through major histocompatibility complex-I (MHC-I) to cluster of differentiation 8+ (CD8^+^) T cancerous cells which ultimately leads to antitumor immune response and the lysis of cancerous cells through the release of granzyme B, perforin and interferon-gamma (IFN-γ) (Keshavarz et al., 2019).

The paramyxoviruses (PVs) can be described as viruses causing diseases in both humans and animals and belong to the members of the Paramyxoviridae. They are non-segmented negative-sense RNA viruses with envelopes and a diameter of 100–300 nm (Javaheri et al., 2022). Examples of PV include morbillivirus, measles, Newcastle disease virus, and so on (Keshavarz et al., 2019). A polycistronic gene that encodes two or more overlapping open reading frames (ORFs) is a popular genetic feature shared by viruses belonging to the paramyxoviruses (Horvath, 2004). Oncolytic PVs have an impressive affinity to cancerous cells that have viral receptors on their surface. For example, cancer cells with overexpression of sialoglycoproteins receptors can be highly and selectively bonded by NDV and MuV (Matveeva et al., 2018). Various danger signals can be activated by the PVs to establish excellent anti-cancer innate and adaptive immune responses (Figure 6). PVs are strong inducers of interferons (IFN) and other immuno-stimulating cytokines. A great advantage of PVs is the ability to trigger syncytium formation (Matveeva et al., 2015). T cell targeted therapy is becoming popular in clinical cancer treatment. The single chain variable fragments (scFv) of two antibodies of the bispecific T cell engagers (BiTEs) can be used to channel T cells to destroy target cells (Huang et al., 2021). A great example was the genetic engineering of the measles virus with BiTEs measles virus encoding bispecific T cell engagers (MV-BiTEs) and tested against solid tumors. *In vitro* models and *in vivo* models were both tested to determine the oncolytic functionality of MV-BiTEs. MV engineering with BiTE cassettes did not attenuate the oncolytic or replicative efficacy against solid tumors nor did it induce toxicity. The functionality is based on the ability to bind to antigens, specificity in T cell activation, and inducing T cell cytotoxicity. The delivery of some oncolytic agents remains a challenging area and this was reflected with MV-BiTEs serum levels being below the serum detection limit between two to 24 h after MV-BiTEs treatment both in syngeneic and patient-derived models. MV-BiTEs can also enable long-term immunity against tumor growths (Speck et al., 2018). Table 4 is a summary of various studies that have applied paramyxoviruses in cancer therapy.

Another interesting oncolytic virus is the mumps virus which has been shown to have a cytopathic effect (CPE). The antineoplastic efficacy of the wild-type of mumps virus had been previously illustrated and it had been revealed that intertumoral administration was better than systemic administration (Asada, 1974; Okuno et al., 1978), but Ammayappan et al. (2016) proved the oncolytic activity of the recombinant MuV-UCs (rMuV-UCs) encoded with human sodium iodide symporter (MV-NIS). In their study, Mumps Virus; Urabe strain (MuV-US) was developed with a reverse genetics platform based on the nucleotide sequence. The recombinant MuV showed a better growth rate than the parent virus. The different infectivity rates observed among the various cell lines can be attributed to a couple of possible factors including various cellular factors, interferon pathway-related genes or receptors and co-receptors expression levels. The colon carcinoma and neuroblastoma cells had significant viral replication while most of the cell lines were not permissive to the MuV-UC mumps virus infection. MuV-UC viruses also had a significant infection in CT-26-LacZ mouse colon carcinoma cells and N2A mouse neuroblastoma cells *in vitro*. It was hypothesized and proven that the green fluorescence protein (GFP) could affect the replication of the virus *in vivo* (Ammayappan et al., 2016). The use of tumor-associated macrophages (TAMs) illustrates a different outlook on the therapeutic role of TAMs in oncolytic virotherapy. This is because TAMs can acquire an anti-tumor phenotype to enhance the anti-tumor effect of the viruses (Tan et al., 2016).

Also, the Newcastle disease virus (NDV) of the PVs class can serve as an oncolytic agent. Wei et al., 2015 genetically engineered a recombinant NDV carrying intact cHAb18 gene (rNDV-18HL). The cHAb18 antibody-engineered NDV served as a new strategy for anti-tumor therapy without attenuating the viral replication of NDV (Wei et al., 2015). Another group discovered members of the avian avulaviruses groups with inherent antitumor activity. Various avian paramyxoviruses (APMVs) were studied to identify the ones with oncolytic capacity. APMV-4 Duck (Hong Kong/D3/1975 OV), new cancer therapeutic was discovered to have greater antitumor properties than the clinical candidate (NDV) and should be clinically translated for the treatment of solid tumors (Javaheri et al., 2022). Studies have shown that NDV is not selectively cytotoxic to normal cells due to a lack in the interferon (IFN) antiviral responses of tumor cells (Elankumaran et al., 2010; Wei et al., 2015; Kazimirsky et al., 2016). Various genetic engineered strains of NDV were studied to understand their potential toxicities and it was observed that the differential regulation of IFN-α and downstream antiviral genes induced by IFN-α determines the tumor-selective replication of rNDV (Elankumaran et al., 2010). Some clinical trials where oncolytic therapy has been applied include the following; attenuated NDV vaccine from the Hertfordshire strain, MTH-68/H clinical trial showed increased survival rate and good quality patient lifestyles (Csatary et al., 2004), and attenuated NDV vaccine from the PV7011 strain also yielded remarkable levels of neutralizing antibodies with signs of tumor regressions after PV701 administration (Pecora et al., 2002).

Among the paramyxoviruses generally, wild-type (WT) parainfluenza virus 5 (PIV5) has been established to poorly induce host cell responses in some human cell types and to be highly non-cytopathic to a lot of *in vitro* cell types. A study showed that the mutated form (P/V-CPI^-^) is highly cytopathic and can destroy HEp-2 human laryngeal cancer cells but some cells might emerge again over time. A proposed solution would be the combination of oncolytic viruses with chemotherapies, and this has found grounds in some ongoing clinical trials. For example, a phase II clinical trial outcome showed that combination therapy of 5-fluorouracil, cisplatin, and oncolytic adenovirus ONYX-015 was more effective than individual therapies in persistent head and neck cancer cases (Fox and Parks, 2018).

## 4 Toxicity and safety concerns surrounding the adoption of oncolytic viruses as immunotherapeutic agents in cancer treatment

Different immunotherapeutic studies with oncolytic viruses have been initiated in the past 10–15 years (Forbes et al., 2018; Torres-Domínguez and McFadden, 2019; Zheng et al., 2019). These studies have taught us a lot about the processes of cancer vaccination and how to choose the best individual oncolytic viruses. To date, just few oncolytic viruses have been authorized for use in cancer therapies, however many more are still in the process of being approved. The approved oncolytic viruses for use against different forms of cancers are described herewith. ECHO-7 (RIGVIR^®^), an unmodified Picornaviridae family virus strain was approved in the Republic of Latvia by the State Agency of Medicines in 2004 to treat skin melanoma, subcutaneous melanoma metastases, and prevent relapse cum metastases upon surgery (Doniņa et al., 2015). ONYX-015 (H101 or Oncorine), a genetically-modified Adenovirus type 5 strain was approved in China by the State Food and Drug Administration in 2005 to treat nasopharyngeal carcinoma (Jiang et al., 2015). Talimogene laherparepvec (T-VEC) or IMLYGIC^®^, a genetically-modified herpes simplex virus I (HSV-1) strain was approved in the United States by the Food and Drug Administration (FDA) in 2015 to treat unresectable cutaneous, subcutaneous and nodal lesions in melanoma patients (Pol et al., 2016). Pelareorep (REOLYSIN^®^), an unmodified reovirus type 3 strain was granted an orphan drug designation to treat malignant glioma, pancreatic, gastric, peritoneal, tube and ovarian cancer in 2015 and fast track designation to treat metastatic breast cancer in 2017, by the United States FDA (Terrível et al., 2020; U.S. Food and Drug Administration, 2022). Also, orphan drug designation was granted to Toca-511 by the United States FDA to treat glioblastoma, but it is still in clinical trial (Rusell and Peng, 2018; Terrível et al., 2020). Some of the oncolytic viruses in clinical trial and awaiting approval include; JX-594 (NCT02630368 for solid tumors, soft tissue sarcoma, breast cancer), DNX-2401 (NCT03896568 for anaplastic astrocytoma, glioblastoma, gliosarcoma, malignant glioma), ColoAd1 or Enadenotucirev (NCT03916510 for locally advanced rectal cancer), GL-ONC1 (NCT05281471 for endometrioid, high-grade serous, platinum-resistant and platinum-refractory ovarian cancer, peritoneal cancer, fallopian tube cancer), H-1PV (NCT01301430 for glioblastoma multiforme; NCT02653313 for carcinoma, pancreatic ductal), ADV-TK (NCT02768363 for prostate cancer; NCT04495153 for non-small cell lung cancer; NCT02446093 for pancreatic adenocarcinoma; NCT03541928 for prostate cancer), Adenovirus/PSA Vaccine (NCT00583024 for refractory prostate cancer) (Terrível et al., 2020; Li et al., 2022; Clinical Trials Database, 2022). However, some oncolytic viruses have been abandoned for cancer therapy due to ineffectiveness, intolerable toxicity and severe safety concerns (Lauer and Beil, 2022).

Few adverse effects and limited fatality have been linked to replicating viruses in many oncolytic viruses used for human trials (Terrível et al., 2020). When compared to the toxicity of standard cytotoxic medicines, the toxicity of oncolytic viruses in viro-immunotherapy is satisfactory. In all, off-target effects and viral mutation/transmission remain the hallmarks of their safety issues (Forbes et al., 2018). The interactions of these engineered oncolytic replicating viruses with both host and environment are far more difficult to predict, because these interactions require oncolytic viruses to be thoroughly structured to have enough virulence needed to significantly decrease and lessen the tumor mass so as to avoid causing adverse effects in a patient receiving such treatment (Shilpa et al., 2014; Reale et al., 2019).

### 4.1 Safety concerns for the host

While it is true that natural and engineered selectivity has reduced pathogenicity, there is still the chance of off-target effects and genetic modification which may result in unintended toxic effects (Hemminki et al., 2020). Viruses have a high possibility of evolving each time the original virus is replicated, resulting in the proliferation of new viral lineages owing to viral polymerase defects. As a result, one major concern with the use of oncolytic viruses is the propensity of these viruses to acquire new cell tropisms or lose restriction factors (Lawler et al., 2017).

According to Lauer and Beil (2022), genetically modified oncolytic viruses were adopted in almost two-thirds of documented oncolytic virus clinical studies rather than natural viruses. Despite the special consideration given to engineered oncolytic viruses and the acceptance of newer oncolytic viruses with low seroprevalence and the ability to avoid neutralizing antibodies, there has been an increased risk of viral spread with less favorable safety profiles documented for the majority of assessed genetically engineered oncolytic viruses (Chaurasiya et al., 2021).

The prospect of oncolytic viruses harboring immunostimulatory genes causing excessive production of immune modulators and, as a result, immune system overreaction, should also be addressed, although such detrimental consequences are yet to be expressively described. In contrast, overexpression of immune responses may improve the success of oncolytic viral treatment, in which viruses that produce immunostimulatory proteins are used (Zeyaullah et al., 2012). Uncontrolled reproduction and viral transmission have been addressed in known oncolytic viruses by including suicide genes (HSV-TK) utilizing drugs; nevertheless, this cannot be confirmed for all oncolytic viruses, particularly newer oncolytic viruses. Incorporating suicide genes (HSV-TK) into drugs to treat viral cells that are out of control may allow the virus to evade immune-mediated elimination, potentially overcoming the immunological barrier to undesirable viral spread and infections (Prestwich et al., 2008; Thomas and Bartee, 2022). Immunity to microbial agents may develop in response to systemic administration because human systems have evolved to combat infections. Serum neutralization and hepatotoxicity are significant challenges in the development of systemic administration. A neutralizing antibody response has been reported in nearly every virus-treated patient, although it has not been linked to a response or shortage thereof (Forbes et al., 2018).

Oncolytic viruses’ toxicity and safety problems may be due to the increased volume necessary to eliminate and prevent relapses of malignant cells. According to Tadesse and Bekuma (2018), it has been challenging to understand how oncolytic viruses travel to the tumor site since most investigations require injecting large viral titers directly into the tumor site. This might have resulted in the serious safety issues mentioned earlier.

### 4.2 Safety concerns for the environment

Following treatment, individuals may shed live replicating viruses, boosting the likelihood of transmission to healthy individuals. Given the fast mutation rate of viruses, particularly RNA viruses, there is a possibility of transmission when released into the environment *via* waste products (Prestwich et al., 2008). For instance, NDV has been isolated from urine for up to 3 weeks following therapy. Although the virus delivered may not be a human pathogen, it may evolve to acquire pathogenic traits and effectively infect the normal host tissues of a victim. Similarly, mutated viruses can revert to wild-type or integrate with wild-type viruses. All these safety issues continue to influence the development of oncolytic viral therapy and its wide acceptance in cancer treatment, even with their potency as immunotherapeutic agents.

## 5 Future perspectives

Over the years, the usage of oncolytic viruses as a sole anti-cancer agent both at the preclinical and clinical stages has proven effective, but with some limitations emerging as a result of the dynamic nature of tumor cells. There is possibility for the cancer to reoccur and metastasize after being treated with the oncolytic virus. As with the case of HSV-1716 (Seprehvir) which was not recovered from the tumor after administration, there is need to establish the factors that contribute to the biodistribution of oHSV to aid in targeted therapy against metastatic tumors. Since oncolytic viruses stimulate both the innate and adaptive immune system, efforts need to be channeled towards the adequate understanding of the interaction between the oncolytic virus, the tumor and the immune system of the host. This will help in creating better strategies, based on the findings of these studies, to fight against various cancer cells.

Researchers need to focus on exploring other types of unexplored viruses, but with oncolytic property, particularly in clinical trials. These viruses will serve as alternative viruses when there is diminished efficacy of a particular oncolytic virus as a result of repetitive administrations. It has become very clear that there is need for synergistic therapy of oncolytic viruses with other cancer therapies. As highlighted in this review, there are already studies combining oncolytic viro-immunotherapy with other methods like chemotherapy and radiotherapy, but this space is yet to be fully maximized. An example is the better outcome achieved with 5-fluorouracil, cisplatin, and oncolytic adenovirus ONYX-015 in head and neck cancer clinical trial phase II than observed when the oncolytic adenovirus ONYX-015 was applied (Fox and Parks, 2018). When vvDD-IL-12-FG was combined with a PD-1 inhibitor, it radically increased the survival of the model mice used. This promising synergistic therapy also needs to proceed to clinical trial phases (Ge et al., 2020). In order to maximize this combinational approach, researchers need to put some factors into consideration; the mode/method and route of delivery (intratumoral, intraneoplastic, intravenous, intracranial, intraperitoneal, intracarotid, intracerebral, intracavitary, intradermal, peritumoral, inhalation, among others), location of the cancer cells, toxicity to non-tumor healthy cells, tissues or organs of the host, specificity in selection of dosage/titer and the specific immune responses elicited. This will greatly empower the arsenal of oncolytic viruses as immunotherapeutic agents against different types of cancer, and appreciably lessen the global burden of the disease.

## 6 Conclusion

The efficacy of oncolytic viruses as immunotherapeutic agents for the treatment of different forms of cancers has been well established with improvement in their safety, production, selectivity, potency and methods of delivery. It is interesting to know that oncolytic viruses do not only lyse cells, but can also stimulate different types of immune responses, depending on the group of virus used as the oncolytic agent. It is more interesting to know that the genetic activities of these oncolytic viruses could be modified or manipulated to prevent these viruses from being killed by the cells of the host immune system and to enhance their therapeutic potential against tumor cells. In the future, the success of oncolytic viruses in cancer immunotherapy will depend largely on approaches to combine them with conventional cancer therapies and to understand the specific interaction between the tumor and immune status of individual patients.
